# Prevalence of iron deficiency and its association with cardiac function in dogs with various stages of myxomatous mitral valvular disease

**DOI:** 10.3389/fvets.2026.1783638

**Published:** 2026-04-17

**Authors:** Arata Kitazawa, Ryohei Suzuki, Yunosuke Yuchi, Shuji Satomi, Shimpei Kawai, Takahiro Teshima, Hirotaka Matsumoto

**Affiliations:** Laboratory of Veterinary Internal Medicine, School of Veterinary Science, Faculty of Veterinary Medicine, Nippon Veterinary and Life Science University, Musashino-shi, Tokyo, Japan

**Keywords:** anemia, echocardiography, high-output heart failure, iron metabolism, systemic vascular resistance

## Abstract

**Introduction:**

Iron deficiency (ID) is known to increase cardiac workload and contribute to cardiac dysfunction in humans. However, its prevalence in canine heart disease remains poorly understood.

**Methods:**

This retrospective cohort study aimed to determine the prevalence of ID in dogs with myxomatous mitral valvular disease (MMVD) and evaluate its association with cardiac function. Dogs diagnosed with MMVD at the Veterinary Teaching Hospital of Nippon Veterinary and Life Science University between August 2020 and December 2025 were retrospectively evaluated. ID was assessed using transferrin saturation (TSAT) as a marker of iron metabolism. Dogs were stratified into three groups according to TSAT levels. Data from physical examinations, hematological and biochemical analyses, and echocardiographic assessments were analyzed. Statistical analyses were conducted to evaluate intergroup differences and correlations between TSAT levels and clinical variables.

**Results:**

ID was identified in 12% (9/84) of the dogs. Dogs in the ID group demonstrated significantly higher stroke volume and cardiac output compared with those in the normal-TSAT group. In addition, TSAT was significantly and inversely correlated with cardiac output.

**Discussion:**

These findings indicate that ID is present in a subset of dogs with MMVD and is associated with increased cardiac output, likely reflecting reduced systemic vascular resistance. This hemodynamic alteration may represent a potential risk factor for the development of high-output heart failure in this population.

## 1 Introduction

Iron is an essential trace element required for the maintenance of physiological homeostasis (1, 2). Its primary function is oxygen transport (3). Iron deficiency (ID) is characterized by the depletion of iron stores and may ultimately result in ID-related anemia and other metabolic disturbances (3). The development of ID may be attributed to multiple factors, including malnutrition associated with cachexia, bleeding, gastrointestinal disorders, renal disease, and cardiac disease (2, 4, 5). In human medicine, ID has been reported in approximately 40–60% of patients with chronic heart disease, even in the absence of anemia or overt hematological abnormalities (6). As ID adversely affects prognosis (7) and is associated with impaired cardiac function (8), its association with cardiac disease has gained considerable attention. Consequently, the therapeutic efficacy of iron supplementation in patients with cardiac disease is currently under investigation (9). Several methods are available for assessing ID. In human medicine, bone marrow biopsy is considered the gold standard for evaluating iron metabolism (4). However, owing to its invasiveness and potential for adverse effects, this approach has largely been replaced in clinical practice by the measurement of circulating biomarkers. Ferritin, an acute phase protein, primarily binds to iron in tissues and serves as a marker of iron stores (10). However, ferritin levels may be artificially elevated in the presence of inflammation (11). Transferrin saturation (TSAT) represents an alternative marker of iron availability. TSAT, defined as the percentage of transferrin bound to iron, reflects the amount of circulating iron accessible to metabolically active cells (12). Recent studies have demonstrated that TSAT alone is independently associated with all-cause and cardiovascular mortality in the general human population (13, 14).

In veterinary medicine, ID has also been recognized, and its pathological characteristics are considered comparable to those observed in humans (15). A previous study reported that decreased hemoglobin levels were associated with an increased risk of cardiac-related mortality in dogs with myxomatous mitral valvular disease (MMVD) (16). As iron is an essential structural component of hemoglobin, ID may contribute to decreased hemoglobin levels in affected dogs. Accordingly, the evaluation of iron metabolism in dogs with cardiac disease may have important clinical implications. Furthermore, a comprehensive understanding of the pathophysiology of ID and the implementation of appropriate management strategies are essential (17). Nonetheless, no standardized criteria or consensus definitions currently exist for ID or iron overload in dogs (18–21).

Accordingly, the present study aimed to determine the prevalence of ID in dogs with cardiac disease and evaluate its association with cardiac function. ID was postulated to be relatively common in dogs with MMVD, the most prevalent cardiac disease in this species, and to be associated with cardiac function. Additionally, ID was considered a potential contributor to cardiac dysfunction in dogs with MMVD as well as in humans.

## 2 Materials and methods

### 2.1 Study protocol and animals

This retrospective cohort study was approved by the ethics committee of Nippon Veterinary and Life Science University (approval number: R2-5, 25–09). The study period spanned from August 2020 to December 2025, and all enrolled dogs were treated at Veterinary Teaching Hospital of Nippon Veterinary and Life Science University. Dogs diagnosed with MMVD that underwent a comprehensive physical examination, blood testing, and echocardiographic evaluation were included in the study. Dogs with MMVD were stratified into stage B1, B2, and C/D groups according to the American College of Veterinary Internal Medicine (ACVIM) consensus guidelines (22). Pulmonary hypertension (PH) was diagnosed based on an echocardiographic probability, in accordance with the ACVIM consensus statement (22). Dogs were classified as having PH when an intermediate or high probability was identified. Cachexia was defined by the presence of evident anorexia and a weight loss exceeding 5% over the past 6 months (23). Dogs with concurrent malignant neoplasia, dehydration, or inflammatory conditions were excluded owing to their potential impact on cardiovascular hemodynamics and iron metabolism.

Dogs were classified into three groups according to TSAT levels defined on the basis of previous studies in humans and dogs, and variables were compared among these groups. Dogs with a TSAT level of <20% were classified as the ID group, those with TSAT levels of 20–50% as the normal-TSAT group, and those with a TSAT level of >50% as the high-TSAT group (3, 24, 25). As serum ferritin concentrations were available only in a subset of dogs, an additional ferritin-based classification was applied for secondary analyses. Specifically, dogs with a serum ferritin concentration of <89 ng/mL were also classified as the ID group for these analyses (26). The results from the secondary analyses are presented in Supplementary Tables 1–5.

### 2.2 Blood tests

Blood test data were obtained from complete blood counts (Celltac *α*, MEK-6550; Nihon Kohden, Tokyo, Japan) and blood biochemical analyses (BioMajesty, JCA-BM6050; JEOL, Tokyo, Japan) performed at the medical center. All analyses were conducted on the same day as blood sampling. The evaluated parameters included red blood cell (RBC) count, hematocrit, hemoglobin level, mean corpuscular volume (MCV), blood urea nitrogen (BUN) level, creatinine level, serum iron level, total iron-binding capacity (TIBC), and TSAT level. Hemoglobin was used as an indicator of anemia, which was defined as a hemoglobin level of <12.2 g/dL (27). Serum iron concentration was considered to reflect the amount of iron bound to transferrin in the blood. Transferrin saturation was calculated using the following formula: TSAT(%)=iron(μg/dL)/TIBC(μg/dL)×100 (14). In the present study, ID was defined as a TSAT level of <20%, in accordance with the reports of previous studies conducted in dogs and humans (3, 13). Serum ferritin concentrations were measured using a latex agglutination immunoassay at a commercial laboratory (FUJIFILM Vet Systems Co., Ltd., Tokyo, Japan), according to the manufacturer’s instructions, using serum samples that were aliquoted and stored at −80 °C on the day of collection. Reference intervals for hematologic and biochemical variables were based on the standard reference values provided by the diagnostic laboratory where the analyses were performed.

### 2.3 Echocardiography

Echocardiographic examinations were performed using a Vivid E95 Ultra Edition system equipped with a 2.9–5.8 MHz transducer (GE Healthcare, Tokyo, Japan). A lead II electrocardiogram was recorded simultaneously, with electrocardiographic tracings displayed during imaging. Data were collected from three consecutive cardiac cycles in sinus rhythm. Image analysis was conducted offline using EchoPAC software (version 204; GE Healthcare). Left atrial enlargement was assessed by calculating the ratio of the left atrial diameter to the aortic root diameter (LA/Ao) (28). The left ventricular (LV) internal dimension at end-diastole was normalized to body weight (kg) raised to the power of 0.294 to evaluate LV enlargement (LVIDDN) (29). Fractional shortening was measured to evaluate cardiac contractile function. All LV parameters were obtained from the right parasternal long-axis view at the level of the cardiac base and papillary muscles (30). LV stroke volume (SV) was calculated using the cross-sectional area method as LV outflow tract cross-sectional area × velocity time integral, which yields volume (cm^3^, equivalent to mL). LV cardiac output (CO) was then calculated as LV SV × heart rate to evaluate LV performance. Spectral Doppler images were acquired from the left apical five-chamber view (31). LV SV index and LV CO index were normalized to body surface area, calculated using the following formula: (body surface area[m^2])=0.101×(body weight[kg])^(2/3) (31). Total SV was calculated as the difference between LV end-diastolic and LV end-systolic volumes derived from the apical four-chamber view using the Simpson’s method of discs and was normalized to body surface area (32). Systemic vascular resistance (SVR) was calculated according to the standard formula: SVR[dyne×s×cm^(−5)]=80×(mean arterial pressure[mmHg]−central venous pressure[mmHg])/CO[L/min] (33). The mean arterial pressure was obtained via indirect blood pressure measurement using a BP-100D II (Fukuda ME, Tokyo, Japan). Central venous pressure was estimated from echocardiographic findings, whereas CO was derived from echocardiographic measurements. For SVR calculations, central venous pressure was assumed to be 5 mmHg when no right atrial enlargement or transvenous distension was observed, as previously described (34, 35).

Right ventricular (RV) function was evaluated by measuring tricuspid annular plane systolic excursion using two-dimensional echocardiography, normalized to body weight (kg) to the power of 0.284 (36). RV fractional area change was also measured and normalized to body weight (37). These measurements were obtained from an RV-focused view and served as indicators of RV systolic function (37). RV SV was calculated using the cross-sectional area method as RV outflow tract cross-sectional area × velocity time integral, which yields volume (cm^3^, equivalent to mL). RV CO was then calculated as RV SV × heart rate to evaluate RV performance (38). RV SV index and RV CO index were normalized to body surface area, similar to LV SV index and CO index (38). Pulmonary vascular resistance (PVR) was calculated according to the standard formula: $\mathrm{PVR}=(\text{tricuspid regurgitation velocity})/(\begin{array}{l} \text{the}\;\mathrm{RV}\;\text{outflow tract time}\\ -\text{velocity integral} \end{array})\;$ (39, 40). PVR was determined only in dogs with measurable tricuspid regurgitation signals.

### 2.4 Two-dimensional speckle tracking echocardiography

Two-dimensional speckle tracking echocardiography was performed to assess myocardial deformation. Left atrial and LV longitudinal strains were obtained from the left apical four-chamber view within the same cardiac cycle. Circumferential strain and strain rate of the LV at the level of the papillary muscles were derived from the right parasternal short-axis view (41). RV longitudinal strain was measured from an RV-focused view using the same cardiac cycles employed for RV morphological and functional assessment, applying LV four-chamber analysis algorithms (42). Only RV free wall strain was analyzed, using a three-segment model tracing from the lateral tricuspid annulus to the RV apex.

All strain values were recorded as the absolute value of the negative peak of the strain waveform (43). Similarly, all strain rate values were recorded as the absolute value of the negative peak of the systolic strain-rate waveform (43).

### 2.5 Statistical analysis

Continuous data were expressed as medians with interquartile ranges. Categorical variables were analyzed using Fisher’s exact tests. The normality of data distribution was assessed using the Shapiro–Wilk test. For comparisons among the three groups, normally distributed data were analyzed using one-way analysis of variance followed by Tukey’s *post-hoc* test, whereas non-normally distributed data were analyzed using the Kruskal–Wallis test followed by the Steel–Dwass test.

Correlation analyses were conducted using Pearson’s correlation for parametric variables and Spearman’s correlation for non-parametric variables.

A *p* < 0.05 was considered significant. Effect sizes were calculated for both the Kruskal–Wallis test (ε^2^) and *post hoc* Steel–Dwass comparisons (r). All statistical analyses were performed using R software (version 2.8.1; The R Foundation, Vienna, Austria).

## 3 Results

Dogs with MMVD were categorized into three groups based on TSAT levels (Tables 1–5). Comparisons among the three groups, classified by TSAT and ferritin levels, are presented in Supplementary Tables 1–5.

**Table 1:** Physical examination findings, MMVD classification, and historical data for three groups of dogs with MMVD, stratified by TSAT levels.

Variable, unit (summary format)	Overall	ID (*n* = 9)	Normal-TSAT (*n* = 63)	High-TSAT (*n* = 12)	*p-*value
Age, years [median (IQR)]	11.8 (9.3–13.2)	11.5 (9.3–12.1)	12.2 (9.9–13.9)	10.4 (8.0–12.5)	0.26
Sex (male, female, cast, spay) [n (%)]	15, 7, 30, 32 (18, 8, 36, 38)	1, 0, 7, 1 (11, 0, 78, 11)	12, 7, 18, 26 (19, 11, 29, 41)	2, 0, 5, 5 (17, 0, 42, 42)	0.20
Body weight, kg [median (IQR)]	4.6 (3.3–6.8)	5.0 (4.0–6.4)	4.9 (3.3–7.0)	3.6 (3.0–4.1)	0.10
Heart rate, bpm [median (IQR)]	131 (116–147)	140 (128–150)	134 (123–152)	124 (118–139)	0.56
Systolic blood pressure, mmHg [median (IQR)]	137 (129–149)	141 (137–144)	137 (129–148)	139 (127–160)	0.98
ACVIM (B1, B2, and C/D) [n (%)]	29, 31, 24 (35, 37, 29)	6, 1, 2 (67, 11, 22)	18, 26, 19 (29, 41, 30)	5, 4, 3 (42, 33, 25)	0.25
PH complications [n (%)]	21 (25%)	2 (22%)	16 (25%)	3 (25%)	0.95
Anemia [n (%)]	6 (7%)	2 (22%)	4 (6%)	0 (0%)	0.16

ACVIM, American College of Veterinary Internal Medicine; ID, iron deficiency; MMVD, myxomatous mitral valvular disease; PH, pulmonary hypertension; TSAT, transferrin saturation.

**Table 2:** Cardiac medication use in dogs with MMVD stratified by TSAT levels.

Variable (summary format)	Overall	ID (*n* = 9)	Normal-TSAT (*n* = 63)	High-TSAT (*n* = 12)	*p-*value
Pimobendan [n (%)]	45 (54%)	4 (44%)	36 (57%)	5 (42%)	0.41
Angiotensin-converting enzyme inhibitor [n (%)]	57 (68%)	4 (44%)	48 (76%)	5 (42%)	0.02
Spironolactone [n (%)]	18 (21%)	3 (33%)	13 (21%)	2 (17%)	0.67
Loop diuretics [n (%)]	19 (23%)	2 (22%)	17 (27%)	0 (0%)	0.10
Amlodipine [n (%)]	14 (17%)	1 (11%)	10 (16%)	3 (25%)	0.61

ID, iron deficiency; MMVD, myxomatous mitral valvular disease; TSAT, transferrin saturation.

**Table 3:** Blood test data in dogs with MMVD stratified by TSAT levels.

Variable, unit (summary format)	Reference intervals	Overall	ID (*n* = 9)	Normal-TSAT (*n* = 63)	High-TSAT (*n* = 12)	*p-*value
RBC, ×10^6^/μL [median (IQR)]	5.7–8.5	6.4 (5.8–7.3)	6.0 (5.1–6.1)	6.7 (6.2–7.4)	7.2 (6.8–7.5)	0.36
Hematocrit, % [median (IQR)]	41–58	45.0 (41.3–50.4)	38.6 (36.2–43.8)	45.0 (41.7–50.0)	51 (47.5–51.2) *	0.03
Hemoglobin, g/dL [median (IQR)]	14.1–20.1	15.2 (13.9–17.0)	12.6 (11.6–14.5)	15.0 (13.9–16.9) *	16.9 (16.1–17.7) *	<0.01
MCV, fL [median (IQR)]	64.0–76.0	66.6 (64.3–68.8)	61.6 (60.8–67.4)	66.8 (64.9–68.8)	67.3 (65.6–69.0)	0.36
BUN, mg/dL [median (IQR)]	9.2–29.2	25.3 (17.8–42.3)	32.8 (22.3–41.4)	25.5 (19.3–45.7)	18.7 (15.4–31.4)	0.23
Creatinine, mg/dL [median (IQR)]	0.40–1.40	0.9 (0.8–1.2)	0.9 (0.7–1.3)	1.0 (0.8–1.2)	0.8 (0.6–1.0)	0.23
Serum iron, μg/dL [median (IQR)]	102–304	130 (99–168)	83 (59–96)	123 (106–156) *	229 (178–264) ***	<0.01
TIBC, μg/dL [median (IQR)]	284–515	401 (339–474)	465 (346–489)	410 (358–476)	314 (293–358) **	<0.01
TSAT, % [median (IQR)]	20–50	31.0 (24.4–41.2)	17.1 (15.8–18.5)	30.9 (25.6–36.5)	71.9 (59.7–76.8)	-
Serum ferritin, ng/mL [median (IQR)]	46–231	161 (105–257)	129 (117–241)	161 (105–229)	189 (132–274)	-

*n* = 84 unless otherwise indicated; ferritin was measured in 30 dogs (ID: *n* = 3, normal-TSAT: *n* = 24, high-TSAT: *n* = 3). Reference intervals are based on the standard values provided by the diagnostic laboratory. *p < 0.05 vs. ID group. **p < 0.05 vs. normal-TSAT group. ***p < 0.05 vs. ID and normal-TSAT groups. BUN, blood urea nitrogen; ID, iron deficiency; MCV, mean corpuscular volume; MMVD, myxomatous mitral valvular disease; RBC, red blood cell; TIBC, total iron-binding capacity; TSAT, transferrin saturation.

**Table 4:** LV variables assessed by echocardiography in dogs with MMVD stratified by TSAT levels.

Variable, unit (summary format)	Reference value/interval	Overall	ID (*n* = 9)	Normal-TSAT (*n* = 63)	High-TSAT (*n* = 12)	*p*-value
LA/Ao, − [median (IQR)]	<1.6	1.8 (1.5–2.2)	1.7 (1.5–1.8)	1.9 (1.5–2.2)	1.8 (1.4–2.2)	0.70
LVIDDN, − [median (IQR)]	≤1.7	1.7 (1.5–2.0)	1.7 (1.5–2.0)	1.7 (1.6–2.1)	1.8 (1.6–1.9)	0.79
Fractional shortening, % [median (IQR)]	25–45	52.4 (44.0–57.6)	57.8 (42.4–60.6)	51.7 (44.2–56.9)	55.1 (44.2–56.4)	0.69
LV SV index, mL/m^2^ [median (IQR)]	Method-dependent	21.6 (15.9–27.6)	33.3 (23.2–36.1)	21.0 (15.4–24.9) *	25.1 (13.4–30.7)	0.02
LV CO index, L/min/m^2^ [median (IQR)]	Method-dependent	2.6 (2.2–3.6)	4.0 (3.3–5.4)	2.6 (2.0–3.3) *	3.0 (1.8–3.7)	0.03
Total SV, mL/m^2^ [median (IQR)]	Method-dependent	36.8 (25.6–46.5)	35.3 (33.4–43.8)	37.2 (24.9–48.0)	36.9 (26.8–45.0)	0.94
SVR, dyne×s × cm^−5^ [median (IQR)]	No universally established RI	2,843 (2,210–3,552)	1,826 (1,386–2,486)	2,871 (2,381–3,621) *	3,132 (2,316–4,605)	0.03
LV longitudinal strain, % [median (IQR)]	Published reference values vary	20.8 (17.9–25.2)	21.2 (19.8–21.5)	21.0 (18.3–25.2)	18.8 (15.9–24.5)	0.81
LV longitudinal strain rate, %/s [median (IQR)]	Published reference values vary	3.0 (2.3–3.7)	2.9 (2.5–3.5)	3.0 (2.3–3.6)	3.0 (2.3–4.2)	0.62
LV circumferential strain, % [median (IQR)]	Published reference values vary	23.3 (19.1–26.1)	25.2 (18.0–27.5)	22.6 (19.0–25.4)	25.4 (22.5–28.2)	0.32
LV circumferential strain rate, %/s [median (IQR)]	Published reference values vary	2.9 (2.1–3.7)	2.7 (2.5–3.7)	2.9 (2.0–3.6)	3.2 (2.6–3.8)	0.38

*p < 0.05 vs. ID group. ID, iron deficiency; LA/Ao, the left atrial diameter to the aortic root diameter; LV CO index, left ventricular cardiac output normalization; LV SV index, left ventricular stroke volume normalization; LVIDDN, the left ventricular internal dimension at end-diastole normalization; MMVD, myxomatous mitral valvular disease; SV, stroke volume; SVR, systemic vascular resistance; TSAT, transferrin saturation.

**Table 5:** RV variables assessed by echocardiography in dogs with MMVD stratified by TSAT levels.

Variable, unit (summary format)	Reference value/interval	Overall	ID (*n* = 9)	Normal-TSAT (*n* = 63)	High-TSAT (*n* = 12)	*p-*value
Tricuspid regurgitation, m/s [median (IQR)]	–	2.9 (2.6–3.3)	3.0 (2.5–3.5)	2.9 (2.6–3.2)	2.8 (2.7–3.2)	0.95
Tricuspid annular plane systolic excursion index, mm [median (IQR)]	≥ 4.5	11.9 (10.5–13.4)	12.1 (11.0–13.2)	11.8 (10.5–13.2)	11.6 (10.5–14.3)	0.83
RV fractional area change index, % [median (IQR)]	>30.0	55.6 (45.9–62.4)	54.7 (50.7–65.6)	55.7 (45.9–63.0)	51.0 (44.5–57.1)	0.44
RV SV index, mL/m^2^ [median (IQR)]	Method-dependent	26.6 (22.0–34.2)	33.3 (23.2–36.1)	26.4 (21.6–32.9) *	25.1 (13.4–30.7)	0.02
RV CO index, L/min/m^2^ [median (IQR)]	Method-dependent	3.5 (2.5–4.4)	4.0 (3.3–5.4)	3.5 (2.5–4.1) *	3.0 (1.8–3.7) *	<0.01
PVR [median (IQR)]	No universally established RI	0.3 (0.3–0.4)	0.2 (0.2–0.2)	0.3 (0.3–0.4) *	0.4 (0.3–0.5) *	<0.01
RV longitudinal strain, % [median (IQR)]	Published reference values vary	26.5 (23.1–35.0)	27.5 (23.0–36.9)	26.8 (23.5–35.8)	24.1 (21.2–25.2)	0.12
RV longitudinal strain rate, %/s [median (IQR)]	Published reference values vary	2.9 (2.1–4.1)	2.4 (1.9–3.5)	3.0 (2.2–4.7)	2.4 (2.2–3.1)	0.24

*p < 0.05 vs. ID group. ID, iron deficiency; MMVD, myxomatous mitral valvular disease; PVR, pulmonary vascular resistance; RV CO index, right ventricular cardiac output normalization; RV SV index, right ventricular stroke volume normalization; TSAT, transferrin saturation.

### 3.1 Historical data and physical examinations

The physical examination findings and historical data of the three groups are presented in Table 1. No significant differences were observed between the groups in age, sex, body weight, heart rate, systolic blood pressure, ACVIM severity classification, or the presence of PH and anemia. Cachexia was not observed in any dog. Reported cardiovascular complications included tricuspid regurgitation, PH, and aortic regurgitation. Tricuspid regurgitation was identified in 59 dogs, of whom 23 also had PH, whereas AR was present in 5 dogs. Based on retrospective echocardiographic data, none of the dogs showed evidence of right-sided heart failure or marked enlargement of the right atrium or hepatic veins, and therefore central venous pressure was estimated to be 5 mmHg in all cases.

A total of 84 dogs with MMVD were included in the study, comprising 17 Chihuahuas, 13 Toy Poodles, 10 mixed-breed dogs, 9 Pomeranians, 8 Cavalier King Charles Spaniels, 6 Maltese dogs, 4 Miniature Dachshunds, 3 Miniature Schnauzers, 2 Shih Tzus, 2 golden retrievers, 2 Shetland Sheepdogs, 2 Italian greyhounds, and 1 dog each of Cairn Terrier, Dachshund, Boston Terrier, Yorkshire Terrier, Chinese Crested, and Shiba Inu.

Cardiovascular medications administered in each group are presented in Table 2. A significant association was observed between angiotensin-converting enzyme inhibitor use and group classification. Specifically, angiotensin-converting enzyme inhibitors were administered in 76% of dogs in the normal-TSAT group, compared with 44% in the ID group and 42% in the high-TSAT group.

For the secondary analysis, two dogs initially classified within the normal-TSAT group were reclassified into the ID group based on TSAT and ferritin levels. Physical examination findings and historical data for the three groups, classified according to TSAT and ferritin levels, are summarized in Supplementary Table 1. A significant association was observed among the groups in age. No significant associations were observed among the groups in the other indices. Cardiovascular medications administered to the three groups based on TSAT and ferritin levels in dogs with MMVD are summarized in Supplementary Table 2. No significant association was observed between the groups.

### 3.2 Blood tests

Blood test data compared across three groups based on TSAT levels in dogs with MMVD are presented in Table 3. Significant differences were observed between the groups in hematocrit (ω^2^ = 0.07), hemoglobin (ε^2^ = 0.12), serum iron (ω^2^ = 0.39), and TIBC (ε^2^ = 0.10). Serum ferritin was measured in 30 dogs. Among them, three dogs exhibited ferritin levels < 89, and two were subsequently reclassified from the normal-TSAT group to the ID group in the secondary analysis. Blood test data compared across the three groups based on TSAT and ferritin levels in dogs with MMVD are summarized in Supplementary Table 3. In the ID group, a significant association was observed in serum iron levels compared with the other groups.

### 3.3 Echocardiographic variables for the left ventricle

**Figure 1:**
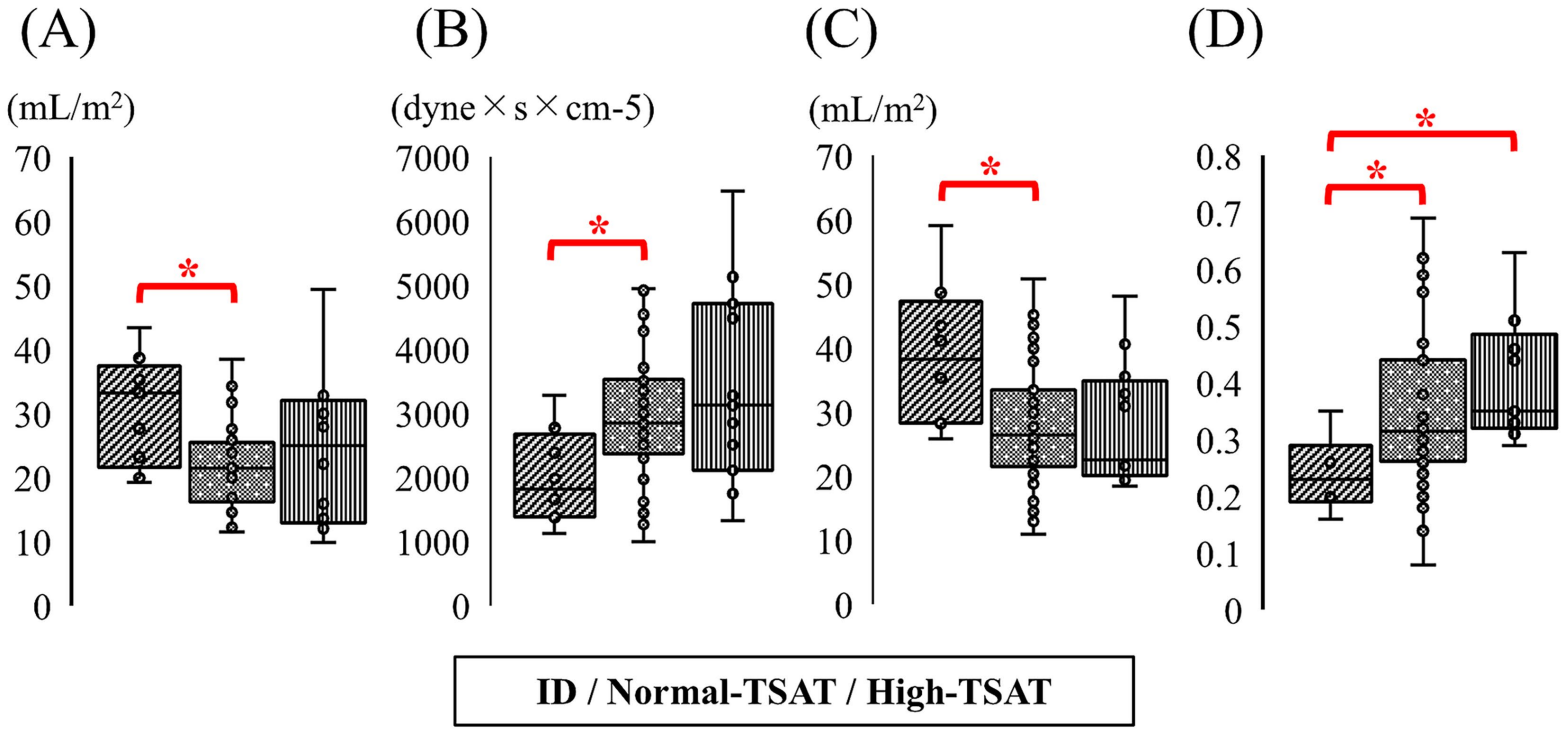
Box plots comparing the hemodynamic variables among the three groups of dogs with MMVD stratified by TSAT levels. **(A)** Box plots comparing the LV SV index. **(B)** Box plots comparing the SVR. **(C)** Box plots comparing the RV SV index. **(D)** Box plots comparing the PVR. *p < 0.05. ID, iron deficiency; LV SV index, left ventricular stroke volume index; MMVD, myxomatous mitral valvular disease; PVR, pulmonary vascular resistance; RV SV index, right ventricular stroke volume index; SVR, systemic vascular resistance; TSAT, transferrin saturation.

Variables of LV function obtained from echocardiography for the three groups classified by TSAT levels in dogs with MMVD are presented in Table 4. Significant differences were observed among the groups in LV SV index (ω^2^ = 0.10), LV CO index (ε^2^ = 0.06), and SVR (ε^2^ = 0.06). Pairwise comparisons indicated that the ID group had a significantly higher LV SV index compared with the normal-TSAT group (*p* = 0.02, r = 0.72, 95% confidence interval (CI): 0.48–0.87) (Figure 1A). Pairwise comparisons demonstrated that the ID group had a significantly higher LV CO index compared with the normal-TSAT group (*p* = 0.02, *r* = 0.31, 95% CI: 0.12–0.55). Pairwise comparisons indicated that the ID group had a significantly lower SVR compared with the normal-TSAT group (*p* = 0.03, *r* = 0.29, 95% CI: 0.06–0.49) (Figure 1B). No significant differences were observed among the groups in LV morphology, other LV functional parameters or LV speckle-tracking echocardiographic parameters. LV variables obtained from echocardiography for the three groups classified by TSAT and ferritin levels in dogs with MMVD are summarized in Supplementary Table 4. Consistent with the primary analysis, the ID group exhibited significantly increased LV SV index and LV CO index and significantly reduced SVR.

### 3.4 Echocardiographic variables for the right ventricle

RV variables obtained from echocardiography for the three groups classified by TSAT levels in dogs with MMVD are presented in Table 5. Significant differences were observed among the groups in RV SV index (ε^2^ = 0.07), RV CO index (ω^2^ = 0.10), and PVR (ε^2^ = 0.11). Pairwise comparisons indicated that the ID group had a significantly higher RV SV index compared with the normal-TSAT group (*p* = 0.02, *r* = 0.31, 95% CI: 0.09–0.51) (Figure 1C). Pairwise comparisons demonstrated that the ID group had a significantly higher RV CO index compared with the normal-TSAT group (*p* < 0.01, *r* = 0.36, 95% CI: 0.14–0.55). Pairwise comparisons demonstrated that the ID group had a significantly higher RV CO index compared with the high-TSAT group (*p* < 0.01, *r* = 0.57, 95% CI: 0.15–0.82). Pairwise comparisons indicated that the ID group had a significantly lower PVR compared with the normal-TSAT group (*p* = 0.02, *r* = 0.31, 95% CI: 0.09–0.51) and high-TSAT group (*p* < 0.01, *r* = 0.59, 95% CI: 0.19–0.83) (Figure 1D). No significant differences were observed among the groups in other RV functional parameters or RV speckle-tracking echocardiographic parameters. RV variables for the three groups division based on TSAT and ferritin levels are summarized in Supplementary Table 5. Consistent with the primary analysis, the ID group exhibited significantly increased RV SV index and RV CO index and significantly reduced PVR.

### 3.5 Correlation

**Figure 2:**
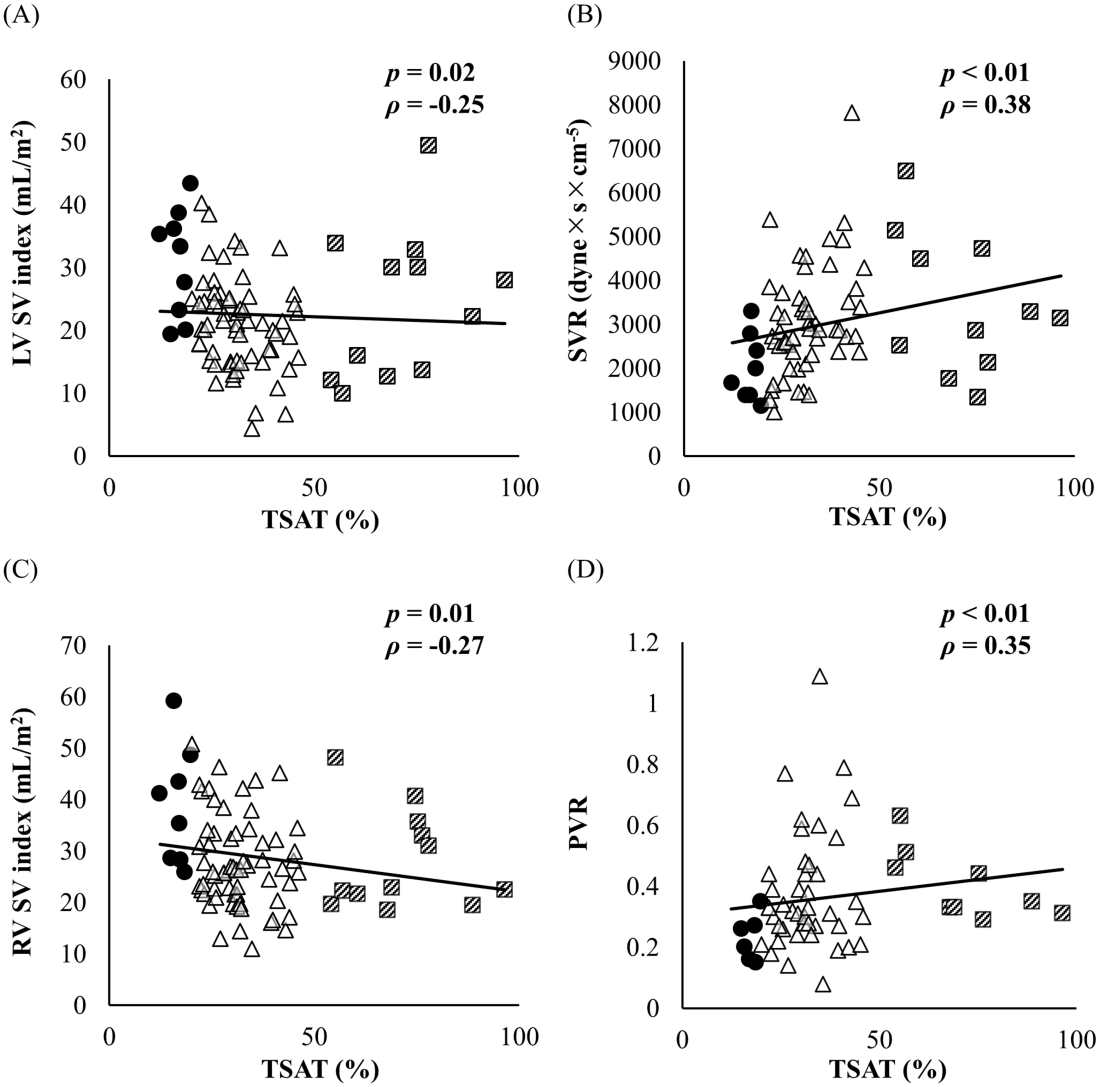
Correlations between TSAT and hemodynamic variables in the dogs with MMVD. Circulars represent dogs in the ID group (*n* = 9), triangles represent dogs in the normal-TSAT group (*n* = 63), and squares represent dogs in the high-TSAT group (*n* = 12). **(A)** Correlation between TSAT and LV SV index. **(B)** Correlation between TSAT and SVR. **(C)** Correlation between TSAT and RV SV index. **(D)** Correlation between TSAT and PVR. ID, iron deficiency; LV SV index, left ventricular stroke volume normalization; MMVD, myxomatous mitral valvular disease; PVR, pulmonary vascular resistance; RV SV index, right ventricular stroke volume normalization; SVR, systemic vascular resistance; TSAT, transferrin saturation.

Hematocrit and hemoglobin levels demonstrated moderate to strong positive correlations with TSAT levels (hematocrit: *ρ* = 0.48, *p <* 0.01, 95% CI: 0.28–0.64; hemoglobin: *ρ* = 0.52, *p <* 0.01, 95% CI: 0.32–0.68). Both LV SV index and LV CO index showed weak to moderate negative correlations with TSAT (LV SV index: Figure 2A) (95% CI: −0.45−−0.03; LV CO index: *ρ* = −0.30, *p <* 0.01, 95% CI: −0.49−−0.09). SVR showed a moderate positive correlation with TSAT (Figure 2B) (95% CI: 0.17–0.56). Similarly, the RV SV index and RV CO index exhibited a weak to moderate negative correlation with TSAT (RV SV index: Figure 2C) (95% CI: −0.44−−0.02; RV CO index: *ρ* = −0.31, *p <* 0.01, 95% CI: −0.50−−0.09). PVR demonstrated a moderate positive correlation with TSAT (Figure 2D) (95% CI: 0.04–0.53).

## 4 Discussion

ID was identified in 12% (9/84) of dogs, indicating that ID may occasionally occur among dogs with MMVD. Although a significant difference in angiotensin-converting enzyme inhibitor use was observed among the groups, the overall medication distribution did not appear sufficient to explain the study findings. Furthermore, no associations were observed between ID and ACVIM stage or indices of cardiac size, implying that the development of ID may be only weakly related to MMVD-associated cardiac disease. This is inconsistent with our original hypothesis. Two potential explanations may account for this discrepancy. First, the underlying causes of ID were not differentiated in this study. ID with normal ferritin is thought to be associated with inflammation and chronic disease and may result from a variety of underlying mechanisms (44). By contrast, ID with decreased ferritin is typically attributed to excessive renal iron loss or impaired gastrointestinal iron absorption (45). Measurement of serum ferritin would allow discrimination between ID with and without decreased ferritin, and such differentiation may be necessary for a more accurate interpretation of the results. Therefore, future studies should include serum ferritin as a routine laboratory parameter for evaluating ID. Second, dogs with severe ID or overt anemia were not included in the present study. Stratification according to the underlying causes of ID may help to identify more detailed associations with cardiac function and morphology.

Hemoglobin levels were lower in dogs with low TSAT and demonstrated a moderate correlation with TSAT. These findings indicate that decreased hemoglobin levels were associated with ID occurrence. Previous studies have identified low hemoglobin levels as a risk factor for cardiac-related death in dogs with MMVD (16), whereas ID has been recognized as a risk factor for cardiac-related mortality in human medicine (4, 6, 7). Therefore, ID may represent a potential risk factor for cardiac-related mortality in dogs with MMVD, warranting further investigation based on the findings of the present study.

No significant differences were observed between the ID group and the other groups in cardiac morphology, functional indices, or detailed myocardial motion analyses. Although previous studies have suggested a potential association between ID and impaired cardiac function, such an association was not evident in the dogs with ID included in this study. These findings suggest that an association between ID and alterations in cardiac morphology or function may become more apparent in dogs with more severe or persistent ID. By contrast, increased SV was observed in both the left and right ventricles (Figures 1A,C, 2A,C). Potential mechanisms underlying the increased SV include medication effects, increased preload, and reduced afterload. Although a significant difference in angiotensin-converting enzyme inhibitor use was observed among the groups, the overall medication distribution did not appear sufficient to explain the increased SV or decreased afterload in the ID group (Table 2). Furthermore, no between-group differences were observed in indices of volume overload, including the left atrium-to-aortic root ratio, or LV internal dimension at end-diastole. SVR, an indicator of afterload, was significantly lower in the ID group (Figures 1B, 2B). Therefore, the observed increase in SV is likely attributable to reduced afterload, potentially reflecting compensatory mechanisms to maintain systemic oxygen delivery in the context of ID. ID may affect RBC-size and hematocrit levels, thereby contributing to a reduction in whole blood viscosity. Decreased blood viscosity may also represent one of the factors underlying the increased SV and reduced SVR observed in this study. Therefore, it is necessary to consider whether our findings reflect a direct response to reduced vascular resistance or are secondary to decreased blood viscosity associated with ID. In the present study, 78% (7/9) of dogs with ID did not have concurrent anemia, suggesting that cases with markedly reduced blood viscosity were likely uncommon in this cohort. However, due to the limited number of dogs with ID-related anemia, we were unable to compare cardiac function and hemodynamic parameters according to the presence or absence of anemia. Future studies including a larger population of dogs with ID-related anemia will be required to evaluate these indices stratified by anemia status, which may help to further differentiate the mechanisms underlying the observed increase in SV.

This hemodynamic profile resembles that of high-output heart failure, a condition in which increased peripheral oxygen demand and reduced SVR lead to excessive increases in SV and venous return (46). Although no dogs in the present study developed high-output heart failure, prolonged or progressive ID may increase venous return and may be associated with the development of high-output heart failure in affected dogs. In cases exhibiting ID and the associated high-output state, iron supplementation therapy may have the potential to suppress excessive increases in CO and alleviate volume overload on the heart. Therefore, further investigation into the therapeutic utility of iron supplementation in dogs with ID is warranted.

In the secondary analysis, the between-group differences in hematocrit and hemoglobin levels observed in the primary analysis were no longer significant between the ID group and the other two groups. By contrast, SV remained significantly higher and SVR remained significantly lower in the ID group, consistent with the findings of the primary analysis. Similar results were observed in the subset of dogs with absolute ID, in which hematocrit, hemoglobin, and TSAT levels were not decreased. These findings indicate that the observed hemodynamic alterations are not solely dependent on TSAT as a single indicator and suggest that a comparable pathophysiological profile may also be present in absolute ID.

In the high-TSAT group, no cases were identified in which hematocrit or hemoglobin values suggested polycythemia. Furthermore, there were no significant differences in cardiac morphology or functional indices between the high-TSAT group and the ID or normal-TSAT groups, and substantial variability was observed within each group. Therefore, in this study, an iron-overload state defined as TSAT > 50% was considered to have minimal impact on hemodynamics.

This study has some limitations. First, the retrospective design may have introduced selection bias and limited control over the completeness and uniformity of clinical data collection. Second, the small sample size may have affected the results, particularly as only a few dogs exhibited excessively low TSAT and anemia, and serum ferritin measurements were available in only a limited number of dogs. Measurement of serum ferritin is necessary to determine the underlying cause of ID; however, this study had limitations in making this differentiation. Third, the measurements of SVR and PVR were based on echocardiographic estimations rather than catheter-based measurements. In particular, the central venous pressure used for the calculation of SVR was estimated based on subjective assessments derived from physical examination and echocardiographic findings, which may have affected the accuracy of the calculated values. Fourth, information regarding the duration of MMVD was not available. Additionally, although not addressed in this study, the severity of valvular regurgitation may have influenced myocardial function and hemodynamics. Fifth, dietary and supplementation information was not documented, limiting the assessment of iron intake as a contributing factor. To address these limitations, future studies should include larger multicenter cohorts, ensure balanced case distribution across MMVD stages, adopt a prospective study design, and systematically record dietary and supplemental intake.

## 5 Conclusion

ID was occasionally observed in dogs with MMVD. Measurement of TSAT enabled the identification of preclinical stages of ID-related anemia. In addition, in dogs with ID, an increase in SV and CO attributable to reductions in SVR and PVR was observed. The progression or long-term persistence of this condition may be associated with the development of high-output heart failure. Therefore, the present study highlights the importance of evaluating ID in dogs with MMVD in future clinical practice.

## Data Availability

The original contributions presented in the study are included in the article/Supplementary material, further inquiries can be directed to the corresponding author.
