# Supplementary material for: Prevalence of iron deficiency and its association with cardiac function in dogs with various stages of myxomatous mitral valvular disease
Source: Front Vet Sci. 2026 Apr 17;13:1783638. doi: 10.3389/fvets.2026.1783638 (PMC13132709; doi:10.3389/fvets.2026.1783638)
Supplement: Supplementary file 1 [file Table_1.docx]

Supplementary Material

# Supplementary Figures and Tables

**Supplemental Table 1.** Physical examination findings, MMVD classification, and historical data for three groups of dogs with MMVD, stratified by TSAT and ferritin levels

| **Variable, unit [summary format]** | **Overall** | **ID (n=11)** | **Normal-TSAT (n=61)** | **High-TSAT (n=12)** | ***p* value** |
| --- | --- | --- | --- | --- | --- |
| **Age, years [median (IQR)]** | 11.8 (9.3–13.2) | 9.6 (7.9–12.0) | 12.2 (10.4–13.9) * | 10.4 (8.0–12.5) | 0.01 |
| **Sex (male, female, cast, spay) [n (%)]** | 15, 7, 30, 32  (18, 8, 36, 38) | 1, 0, 7, 3  (9, 0, 64, 27) | 12, 7, 18, 24  (20, 11, 30, 39) | 2, 0, 5, 5  (17, 0, 42, 42) | 0.54 |
| **Body weight, kg [median (IQR)]** | 4.6 (3.3–6.8) | 4.9 (3.6–5.8) | 5.0 (3.3–7.2) | 3.6 (3.0–4.1) | 0.14 |
| **Heart rate, bpm [median (IQR)]** | 131 (116–147) | 140 (126–149) | 132 (118–147) | 124 (118–139) | 0.10 |
| **Systolic blood pressure, mmHg [median (IQR)]** | 137 (129–149) | 138 (136–143) | 137 (129–149) | 139 (127–160) | 0.87 |
| **ACVIM (B1, B2, and C/D) [n (%)]** | 29, 31, 24  (35, 37, 29) | 7, 3, 1  (63, 27, 9) | 18, 24, 19  (30, 39, 31) | 5, 4, 3  (42, 33, 25) | 0.63 |
| **PH complications [n (%)]** | 21 (25%) | 2 (18%) | 16 (26%) | 3 (25%) | 0.92 |
| **Anemia [n (%)]** | 6 (7%) | 2 (18%) | 4 (7%) | 0 (0%) | 0.27 |

**p* < 0.05 vs. ID group.

ACVIM, American College of Veterinary Internal Medicine; ID, iron deficiency; MMVD, myxomatous mitral valvular disease; PH, pulmonary hypertension; TSAT, transferrin saturation

**Supplemental Table 2.** Cardiac medication use in dogs with MMVD stratified by TSAT and ferritin levels

| **Variable [summary format]** | **Overall** | **ID (n=11)** | **Normal-TSAT (n=61)** | **High-TSAT (n=12)** | ***p* value** |
| --- | --- | --- | --- | --- | --- |
| **Pimobendan [n (%)]** | 45 (54%) | 6 (55%) | 34 (56%) | 5 (42%) | 0.56 |
| **Angiotensin-converting enzyme inhibitor [n (%)]** | 57 (68%) | 6 (55%) | 46 (75%) | 5 (42%) | 0.04 |
| **Spironolactone [n (%)]** | 18 (21%) | 3 (27%) | 13 (21%) | 2 (17%) | 0.83 |
| **Loop diuretics [n (%)]** | 19 (23%) | 3 (27%) | 16 (26%) | 0 (0%) | 0.10 |
| **Amlodipine [n (%)]** | 14 (17%) | 1 (9%) | 10 (16%) | 3 (25%) | 0.64 |

ID, iron deficiency; MMVD, myxomatous mitral valvular disease; TSAT, transferrin saturation

| **Variable, unit [summary format]** | **reference intervals** | **Overall** | **ID (n=11)** | **Normal-TSAT (n=61)** | **High-TSAT (n=12)** | ***p* value** |
| --- | --- | --- | --- | --- | --- | --- |
| **RBC, ×10^6^/ µL [median (IQR)]** | 5.7–8.5 | 6.4 (5.8–7.3) | 6.1 (5.6–7.5) | 6.4 (5.9–7.2) | 7.2 (6.8–7.5) | 0.89 |
| **Hematocrit, % [median (IQR)]** | 41–58 | 45.0 (41.3–50.4) | 41.3 (37.2–49.2) | 44.7 (41.6–49.2) | 51 (47.5–51.2) | 0.12 |
| **Hemoglobin, g/dL [median (IQR)]** | 14.1–20.1 | 15.2 (13.9–17.0) | 14.0 (12.4–15.6) | 14.9 (13.9–16.8) | 16.9 (16.1–17.7) ** | 0.02 |
| **MCV, fL [median (IQR)]** | 64.0–76.0 | 66.6 (64.3–68.8) | 64.4 (61.3–67.3) | 66.6 (64.8–68.8) | 67.3 (65.6–69.0) | 0.36 |
| **BUN, mg/dL [median (IQR)]** | 9.2–29.2 | 25.3 (17.8–42.3) | 26.4 (19.7–38.8) | 25.8 (19.4–46.6) | 18.7 (15.4–31.4) | 0.23 |
| **Creatinine, mg/dL [median (IQR)]** | 0.40–1.40 | 0.9 (0.8–1.2) | 0.7 (0.6–1.2) | 1.0 (0.9–1.2) | 0.8 (0.6–1.0) | 0.10 |
| **Serum iron, µg/dL [median (IQR)]** | 102–304 | 130 (99–168) | 87 (61–104) | 126 (105–158) * | 229 (178–264) *** | <.01 |
| **TIBC, µg/dL [median (IQR)]** | 284–515 | 401 (339–474) | 396 (354–488) | 416 (357–477) | 314 (293–358) *** | <.01 |
| **TSAT, % [median (IQR)]** | 20–50 | 31.0 (24.4–41.2) | 17.4 (16.4–19.3) | 30.9 (25.6–37.4) | 71.9 (59.7–76.8) | - |
| **Serum ferritin, ng/mL [median (IQR)]** | 46–231 | 161 (105–257) | 105 (79–129) | 179 (114–257) | 189 (132–274) | - |

**Supplemental Table 3.** Blood test data in dogs with MMVD stratified by TSAT and ferritin levels

**p* < 0.05 vs. ID group. ***p* < 0.05 vs. normal-TSAT group. ****p* < 0.05 vs. ID and normal-TSAT groups

BUN, blood urea nitrogen; ID, iron deficiency; MCV, mean corpuscular volume; MMVD, myxomatous mitral valvular disease; RBC, red blood cell; TIBC, total iron-binding capacity; TSAT, transferrin saturation

| **Variable, unit [summary format]** | **Reference value / interval** | **Overall** | **ID (n=11)** | **Normal-TSAT (n=61)** | **High-TSAT (n=12)** | ***p* value** |
| --- | --- | --- | --- | --- | --- | --- |
| **LA/Ao,** – **[median (IQR)]** | < 1.6 | 1.8 (1.5–2.2) | 1.7 (1.5–2.1) | 1.9 (1.5–2.1) | 1.8 (1.4–2.2) | 0.82 |
| **LVIDDN,** – **[median (IQR)]** | ≤ 1.7 | 1.7 (1.5–2.0) | 1.8 (1.6–2.2) | 1.7 (1.5–2.0) | 1.8 (1.6–1.9) | 0.83 |
| **Fractional shortening, % [median (IQR)]** | 25–45 | 52.4 (44.0–57.6) | 54.5 (38.5–60.0) | 51.9 (44.5–57.0) | 55.1 (44.2–56.4) | 0.79 |
| **LV SV index, mL/m^2^ [median (IQR)]** | Method-dependent | 21.6 (15.9–27.6) | 27.7 (22.4–35.7) | 21.9 (15.2–25.0) * | 25.1 (13.4–30.7) | 0.02 |
| **LV CO index, L/min/m^2^ [median (IQR)]** | Method-dependent | 2.6 (2.2–3.6) | 3.8 (3.2–4.7) | 2.5 (2.0–3.3) * | 3.0 (1.8–3.7) | 0.02 |
| **Total SV, mL/m^2^ [median (IQR)]** | Method-dependent | 36.8 (25.6–46.5) | 35.3 (32.1–52.3) | 37.2 (24.9–47.5) | 36.9 (26.8–45.0) | 0.85 |
| **SVR, dyne×s×cm^-5^ [median (IQR)]** | No universally established RI | 2,843 (2,210–3,552) | 1,981 (1,456–2,681) | 2,871 (2,416–3,687) * | 3,132 (2,316–4,605) | 0.03 |
| **LV longitudinal strain, % [median (IQR)]** | Published reference values vary | 20.8 (17.9–25.2) | 21.2 (19.5–22.0) | 21.0 (18.3–25.3) | 18.8 (15.9–24.5) | 0.81 |
| **LV longitudinal strain rate, %/s [median (IQR)]** | Published reference values vary | 3.0 (2.3–3.7) | 2.9 (2.4–3.3) | 3.0 (2.3–3.6) | 3.0 (2.3–4.2) | 0.63 |
| **LV circumferential strain, % [median (IQR)]** | Published reference values vary | 23.3 (19.1–26.1) | 21.6 (18.3–26.9) | 23.1 (19.3–25.6) | 25.4 (22.5–28.2) | 0.35 |
| **LV circumferential strain rate, %/s [median (IQR)]** | Published reference values vary | 2.9 (2.1–3.7) | 2.7 (2.4–3.6) | 2.9 (2.0–3.6) | 3.2 (2.6–3.8) | 0.44 |

**Supplemental Table 4.** LV variables assessed by echocardiography in dogs with MMVD stratified by TSAT and ferritin levels

**p* < 0.05 vs. ID group.

ID, iron deficiency; LA/Ao, the left atrial diameter to the aortic root diameter; LV CO index, left ventricular cardiac output normalization; LV SV index, left ventricular stroke volume normalization; LVIDDN, the left ventricular internal dimension at end-diastole normalization; MMVD, myxomatous mitral valvular disease; SV, stroke volume; SVR, systemic vascular resistance; TSAT, transferrin saturation

**Supplemental Table 5.** RV variables assessed by echocardiography in dogs with MMVD stratified by TSAT and ferritin levels

| **Variable, unit [summary format]** | **Reference value / interval** | **Overall** | **ID (n=11)** | **Normal-TSAT (n=61)** | **High-TSAT (n=12)** | ***p* value** |
| --- | --- | --- | --- | --- | --- | --- |
| **Tricuspid regurgitation, m/s [median (IQR)]** | – | 2.9 (2.6–3.3) | 2.7 (2.5–3.4) | 2.9 (2.6–3.4) | 2.8 (2.7–3.2) | 0.85 |
| **Tricuspid annular plane systolic excursion index, mm [median (IQR)]** | ≥ 4.5 | 11.9 (10.5–13.4) | 12.1 (11.3–13.3) | 11.8 (10.3–13.2) | 11.6 (10.5–14.3) | 0.72 |
| **RV fractional area change index, % [median (IQR)]** | > 30.0 | 55.6 (45.9–62.4) | 54.7 (49.7–63.3) | 55.7 (46.0–63.2) | 51.0 (44.5–57.1) | 0.48 |
| **RV SV index, mL/m^2^ [median (IQR)]** | Method-dependent | 26.6 (22.0–34.2) | 28.6 (26.7–42.3) | 26.4 (21.6–33.5) * | 25.1 (13.4–30.7) | 0.13 |
| **RV CO index, L/min/m^2^ [median (IQR)]** | Method-dependent | 3.5 (2.5–4.4) | 4.4 (3.6–5.4) | 3.3 (2.4–4.2) * | 3.0 (1.8–3.7) * | <0.01 |
| **PVR,** – **[median (IQR)]** | No universally established RI | 0.3 (0.3–0.4) | 0.2 (0.2–0.3) | 0.3 (0.3–0.4) | 0.4 (0.3–0.5) * | 0.03 |
| **RV longitudinal strain, % [median (IQR)]** | Published reference values vary | 26.5 (23.1–35.0) | 27.5 (23.2–36.9) | 26.8 (23.4–35.5) | 24.1 (21.2–25.2) | 0.13 |
| **RV longitudinal strain rate, %/s [median (IQR)]** | Published reference values vary | 2.9 (2.1–4.1) | 2.6 (1.9–3.8) | 3.0 (2.1–4.7) | 2.4 (2.2–3.1) | 0.41 |

**p* < 0.05 vs. ID group.

ID, iron deficiency; MMVD, myxomatous mitral valvular disease; PVR, pulmonary vascular resistance; RV CO index, right ventricular cardiac output normalization; RV SV index, right ventricular stroke volume normalization; TSAT, transferrin saturation
